# Methionine as a regulator of bone remodeling with fasting

**DOI:** 10.1172/jci.insight.177997

**Published:** 2024-05-21

**Authors:** Tânia Amorim, Naveen G.V. Kumar, Natalie L. David, William Dion, Trishya Pagadala, Nandini K. Doshi, Bokai Zhu, Andrey Parkhitko, Matthew L. Steinhauser, Pouneh K. Fazeli

**Affiliations:** 1 Aging Institute of UPMC and University of Pittsburgh School of Medicine;; 2 Neuroendocrinology Unit, Division of Endocrinology and Metabolism, Department of Medicine, University of Pittsburgh School of Medicine;; 3 Center for Human Integrative Physiology, Aging Institute of UPMC and University of Pittsburgh School of Medicine;; 4 Division of Endocrinology and Metabolism, Department of Medicine, University of Pittsburgh School of Medicine; and; 5 Division of Cardiology, Department of Medicine, University of Pittsburgh School of Medicine, Pittsburgh, Pennsylvania, USA.

**Keywords:** Endocrinology, Metabolism, Intermediary metabolism, Osteoclast/osteoblast biology, Osteoporosis

## Abstract

Caloric restriction improves metabolic health but is often complicated by bone loss. We studied bone parameters in humans during a 10-day fast and identified candidate metabolic regulators of bone turnover. Pro-collagen 1 intact N-terminal pro-peptide (P1NP), a bone formation marker, decreased within 3 days of fasting. Whereas dual-energy x-ray absorptiometry measures of bone mineral density were unchanged after 10 days of fasting, high-resolution peripheral quantitative CT demonstrated remodeling of bone microarchitecture. Pathway analysis of longitudinal metabolomics data identified one-carbon metabolism as fasting dependent. In cultured osteoblasts, we tested the functional significance of one-carbon metabolites modulated by fasting, finding that methionine — which surged after 3 days of fasting — affected markers of osteoblast cell state in a concentration-dependent manner, in some instances exhibiting a U-shaped response with both low and high concentrations driving putative antibone responses. Administration of methionine to mice for 5 days recapitulated some fasting effects on bone, including a reduction in serum P1NP. In conclusion, a 10-day fast in humans led to remodeling of bone microarchitecture, potentially mediated by a surge in circulating methionine. These data support an emerging model that points to a window of optimal methionine exposure for bone health.

## Abstract 2

Investigation of the effects of fasting on bone metabolism identifies methionine and one carbon metabolism as a potential regulator of fasting-induced bone loss.

## Introduction

Caloric restriction attenuates age-associated diseases and prolongs life span in model organisms (1–5). In humans, short-term studies suggest that caloric restriction and fasting improve metrics of cardiometabolic health in obese, diabetic, and hypertensive patients (6–9). Despite these metabolic benefits, a potential negative consequence of caloric restriction or fasting is decreased bone mineral density (BMD) (10–13). In the CALERIE study, randomization to 24 months of caloric restriction resulted in significant BMD losses at the lumbar spine, total hip, and femoral neck (14), coincident with decreased body weight. CALERIE also reported changes in bone turnover markers, including a significant increase in the bone resorption marker, C-terminal telopeptide of type-I collagen (CTX), and a significant decrease in the bone formation marker alkaline phosphatase (ALPL) (14). With long-term pathological calorie restriction, as seen in women with anorexia nervosa — a psychiatric condition characterized by chronic, inappropriately low caloric intake — low BMD is observed in approximately 85% (15–17), and there is an increased risk of fracture (18–20).

Although bone loss is a known consequence of caloric restriction and low-weight states, the mechanisms and time course of bone loss are not well understood, in part due to technical challenges to mechanistic work. Bone tissue itself is not routinely collected as part of usual patient care, and obtaining bone biopsies as part of research protocols is limited by the invasiveness of the procedure and potential morbidity. Moreover, the extensive requisite sample processing to extract analytes of interest from calcified bone tissue limits the type of information that can be easily retrieved to reflect the in vivo state, even when bone samples are available. This reality has focused attention on identification of circulating markers and determinants of bone metabolism that can be coupled to noninvasive imaging. Dual-energy x-ray absorptiometry (DXA) is commonly used both clinically and for research assessment of areal BMD, but DXA is relatively insensitive to acute changes. By contrast, high-resolution peripheral quantitative computed tomography (HR-pQCT) is a new method to analyze bone microarchitecture that is theoretically more sensitive to assessing bone remodeling over a timescale of relevance to short-term fasting.

The aim of the present study was to first evaluate the effects of an acute fast on parameters of bone microarchitecture with HR-pQCT and second to identify potential regulators of fasting-induced bone remodeling. HR-pQCT demonstrated significant remodeling of bone microarchitecture after 10 days of fasting. We then leveraged metabolomics data to identify one-carbon metabolic pathways and associated metabolites as candidate regulators of bone metabolism with fasting. Finally, we used an in vitro human osteoblast model and murine studies to advance the fasting surge in the one-carbon metabolite, methionine, as a novel regulator of fasting bone metabolism.

## Results

### Bone remodeling with prolonged fasting in healthy humans.

**Figure 1:**
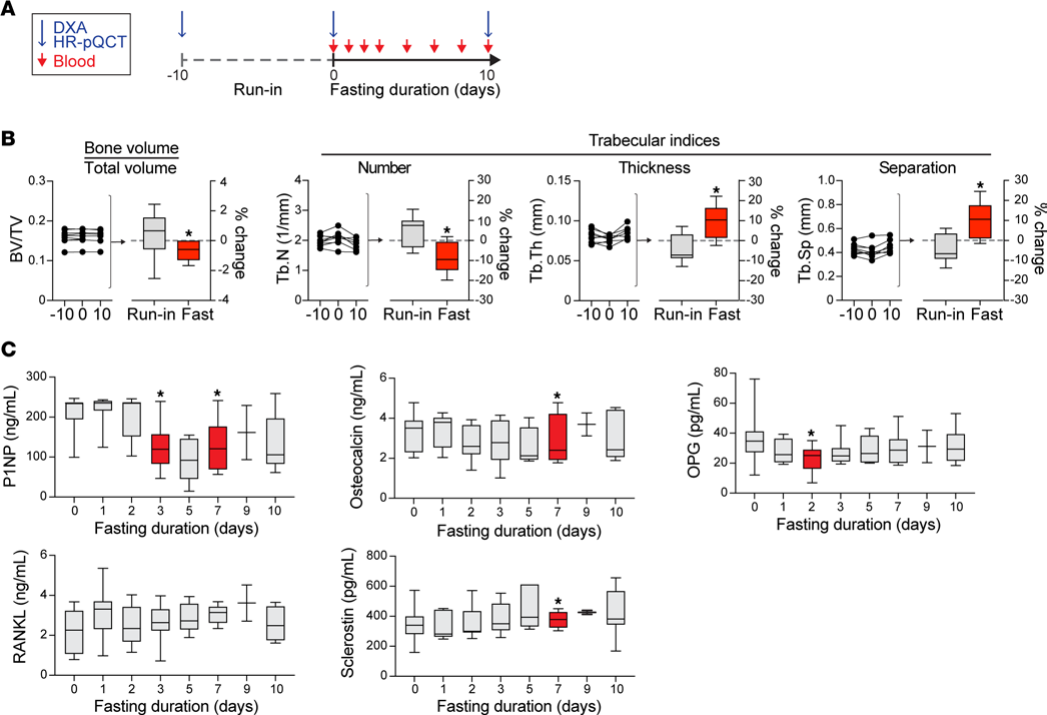
Bone remodeling with prolonged fasting in healthy humans. (**A**) Experimental design. (**B**) High-resolution peripheral quantitative CT (HR-pQCT). Run-in: change in parameters from prebaseline (–10 days) to baseline (day 0). Fast: change in parameters between baseline (day 0) and the conclusion of the fast (day 10); *P < 0.05, paired sample *t* test or paired Wilcoxon signed rank test (if data not normally distributed). (**C**) Bone resorption and formation markers measured by ELISA; *P < 0.05 compared with baseline, paired sample *t* test or paired Wilcoxon signed rank test (if data not normally distributed).

We previously published a 10-day fasting study in healthy humans (21). In this study, we report data on bone parameters in 10 participants (*n* = 7 women) who underwent a 10-day, 0-calorie, inpatient fast (Figure 1A). Characteristics of the study participants at the time of the prebaseline visit are provided in Supplemental Table 1; supplemental material available online with this article; https://doi.org/10.1172/jci.insight.177997DS1 The median BMI for women was 26.5 kg/m^2^ (IQR: 25.2, 28.3) and for men was 28.4 kg/m^2^ (IQR: 27.8, 29.1). Median BMDs at the spine, hip, and distal radius at prebaseline for women and men are shown in Supplemental Table 1. Between the prebaseline visit (approximately 10 days before initiation of the fast) and the baseline visit, there were no significant changes in weight (mean ± SD change in weight between prebaseline and baseline was 0.1% ± 1.7%, *P* = 0.72), BMD, or parameters of bone microarchitecture (Table 1). After 10 days of fasting, however, there was a significant change in bone microarchitecture parameters as assessed by HR-pQCT (Figure 1B and Table 1) coincident with a significant decrease in weight (mean ± SD percentage change in weight: –8.8% ± 1.4%, *P* < 0.0001). Specifically, trabecular number (Tb.N) and BV/TV decreased (% change in Tb.N: –9.2% ± 7.9% mean ± SD, *P* = 0.02; % change in BV/TV: –0.7% ± 0.7%, *P* < 0.05), whereas trabecular thickness (Tb.Th) and separation (Tb.Sp) significantly increased (% change in Tb.Th: 10.3% ± 8.7%, *P* = 0.02; % change in Tb.Sp: 11.0% ± 9.4%, *P* = 0.02) in the radius (Figure 1B). There was a significant increase in cortical density in the radius after 10 days of fasting (% change in cortical density: 0.57% ± 0.52%, *P* = 0.03). By contrast, no significant changes in areal BMD were detected with DXA (Table 1).

We then measured markers of bone formation and resorption, including pro-collagen 1 intact N-terminal propeptide (P1NP), osteocalcin (OCN), CTX, osteoprotegerin (OPG), and receptor activator of nuclear factor kappa beta (RANKL), as well as hormones known to be associated with bone metabolism (sclerostin and parathyroid hormone, PTH) (Figure 1C). P1NP, a marker of bone formation, decreased at the 3-day time point compared with baseline (–37.6% ± 35.7%, *P* = 0.02), remaining low until the conclusion of fasting. Compared with baseline, P1NP levels decreased in 8 of the 9 participants who completed the fast: –36.2% (IQR: –8.3, –65.2). OPG, which is associated with decreased bone resorption, also significantly decreased (% change between baseline and day 2 of fasting: –28.8% ± 37.4%, *P* = 0.04), whereas sclerostin levels significantly increased (+15.7% ± 15.2% at 7 days, *P* = 0.02). Potential markers of bone resorption, CTX and PTH, did not change significantly during the 10-day fast (data not shown). Collectively, these data suggest that anabolic bone formation is attenuated by fasting and in the context of ongoing bone catabolism drives bone remodeling that is detectable over a 10-day period with HR-pQCT.

### Identification of one-carbon metabolites as candidate regulators of anabolic bone metabolism.

**Figure 2:**
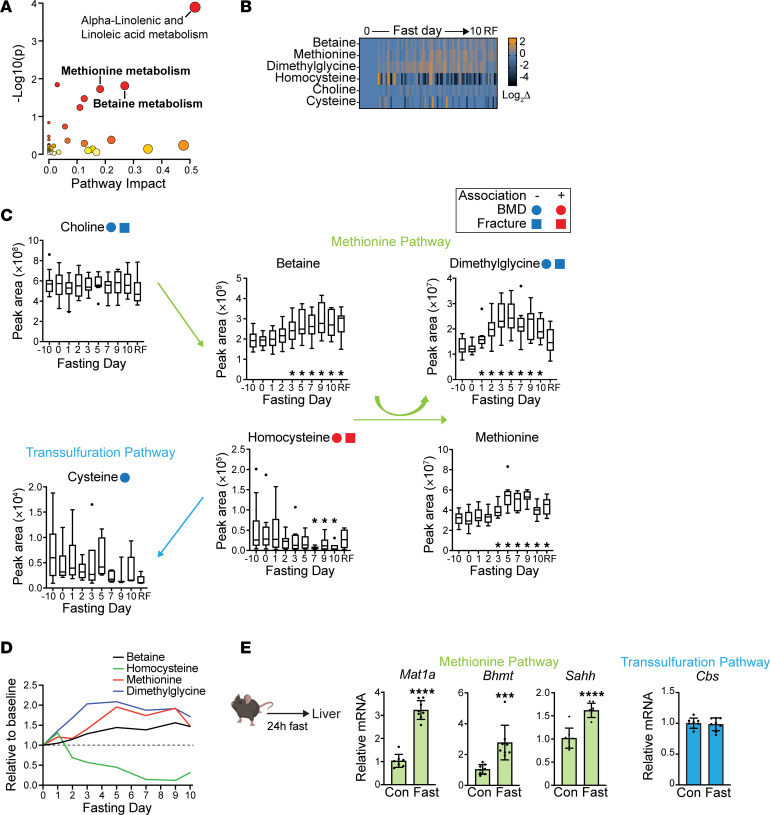
Identification of one-carbon metabolites as candidate regulators of anabolic bone metabolism. (**A**) Pathway analysis using the Small Molecule Pathway Database (SMPDB). (**B**) Heatmap of one-carbon metabolites relative to day 0 of fasting. RF, refeeding. (**C**) One-carbon metabolites with fasting. Associations with bone health from published epidemiological studies. (**D**) Mean one-carbon metabolite levels from **B** expressed relative to baseline (day 0, dashed line). (**E**) Gene expression measured by quantitative PCR (qPCR) of liver in control mice or mice fasted for 24 hours. mRNA expression was normalized to *Gapdh* and to nonfasted control (Con) mice. *P < 0.05, *t* test (*n* = 8 mice per group).

Physiologic perturbations and disease states are often reflected by changes to the circulating metabolome, and specific metabolites may regulate diverse pathobiological processes. Increased plasma glycine and serine, for instance, have been reported in metabolic bone disease states, such as osteoporosis (22). Therefore, we hypothesized that fasting changes in metabolites might play a role in bone remodeling, and our previously published plasma metabolomics analyses of this fasting cohort presented an opportunity to identify novel candidate regulators (21). We first performed a pathway analysis, inputting the subset of metabolites that statistically changed with fasting to the Small Molecule Pathway Database (Figure 2A) (23, 24). The strongest represented pathways were alpha-linolenic and linoleic acid (*P* < 0.001), betaine (*P* < 0.05), and methionine (*P* < 0.05) (Figure 2A). Importantly, the betaine and methionine pathways intersect as part of the same metabolic cycle, implicating one-carbon metabolism, which includes several metabolites that have been either positively or negatively associated with low BMD and risk of fractures in epidemiological studies (25–32), as well as with aging (33).

We next examined one-carbon metabolite dynamics (Figure 2, B–D). Of note, methionine was previously published in graphical form (21); however, we include those data again here to fully contextualize the pathway. Several one-carbon metabolites showed a similar temporal pattern of dynamic increase with fasting, including methionine, betaine, and dimethylglycine, all of which significantly increased with fasting. Interestingly, homocysteine, a metabolite known to be negatively associated with BMD and positively associated with fracture risk (29, 30, 32), decreased over the 10 days of fasting. A related metabolite, cysteine, remained relatively stable with fasting. Taken together, this pattern is consistent with depletion of homocysteine by conversion to methionine (the methionine pathway) with fasting (Figure 2, C and D).

In mammals, the enzyme that drives conversion of homocysteine to methionine with betaine as methyl donor, betaine homocysteine S-methyltransferase (BHMT), is primarily active in the liver (34). Therefore, we examined hepatic expression of genes related to the methionine pathway in a murine fasting model (Figure 2E). The physiology of fasting in mice is temporally compressed with a loss of up to 20% of body mass in 24 hours of fasting and death within days. As such, 24 hours is a common endpoint for murine fasting studies, a time point at which we analyzed transcription of genes involved in the methionine pathway. Compared with nonfasted mice, transcription of *Mat*, *Sahh*, and *Bhmt* was significantly augmented in the livers of fasted mice (*Mat*, *P* < 0.001; *Sahh*, *P* < 0.001; *Bhmt*, *P* < 0.01), whereas transcription of *Cbs* was similar between fasted and nonfasted mice (Figure 2E). Collectively, these data are consistent with upregulation of the methionine metabolism pathway in the liver during fasting as predicted by our human metabolomics fasting data.

### Cultured osteoblasts are sensitive to methionine concentration.

Prior epidemiological evidence linking one-carbon metabolites to metrics of bone health, coupled with rather dramatic shifts in several one-carbon metabolites during the first few days of fasting when circulating markers of anabolic bone formation (P1NP) declined, provided rationale for a new hypothesis: that one-carbon metabolites modulate anabolic bone formation during fasting. We sought to systematically evaluate the subset of one-carbon metabolites that dynamically changed in plasma with fasting. Because P1NP is a marker of anabolic bone formation and the turnover marker that was most sensitive to fasting, we focused our experiments on the cellular source of new bone, the osteoblast. We selected human hFOB1.19 cells as a model for osteoblast differentiation and as a source for human osteoblasts. Even though the cell line is an established osteoblast model, we first verified its differentiation capacity and determined optimal timing for experiments focused on mature osteoblasts. Differentiation was stimulated by the standard protocol of transferring confluent cells from 34°C to 39.5°C. We assessed osteoblast differentiation by alizarin staining, finding robust mineralization matrix by day 16 of differentiation (Supplemental Figure 1A), a time point coinciding with transcription of genes related to mature osteoblast activity, including osteopontin (*OPN*) and bone sialoprotein (*BSP*) (Supplemental Figure 1B). At day 16, cells also responded to 1,25-dihydroxyvitamin D_3_ by increasing expression of *ALPL* and collagen type I alpha 1 chain (*COL1A*) (Supplemental Figure 1C), a characteristic of the mature osteoblast (35, 36). Therefore, we used day 16 cells in subsequent experiments focused on the mature osteoblast.

**Figure 3:**
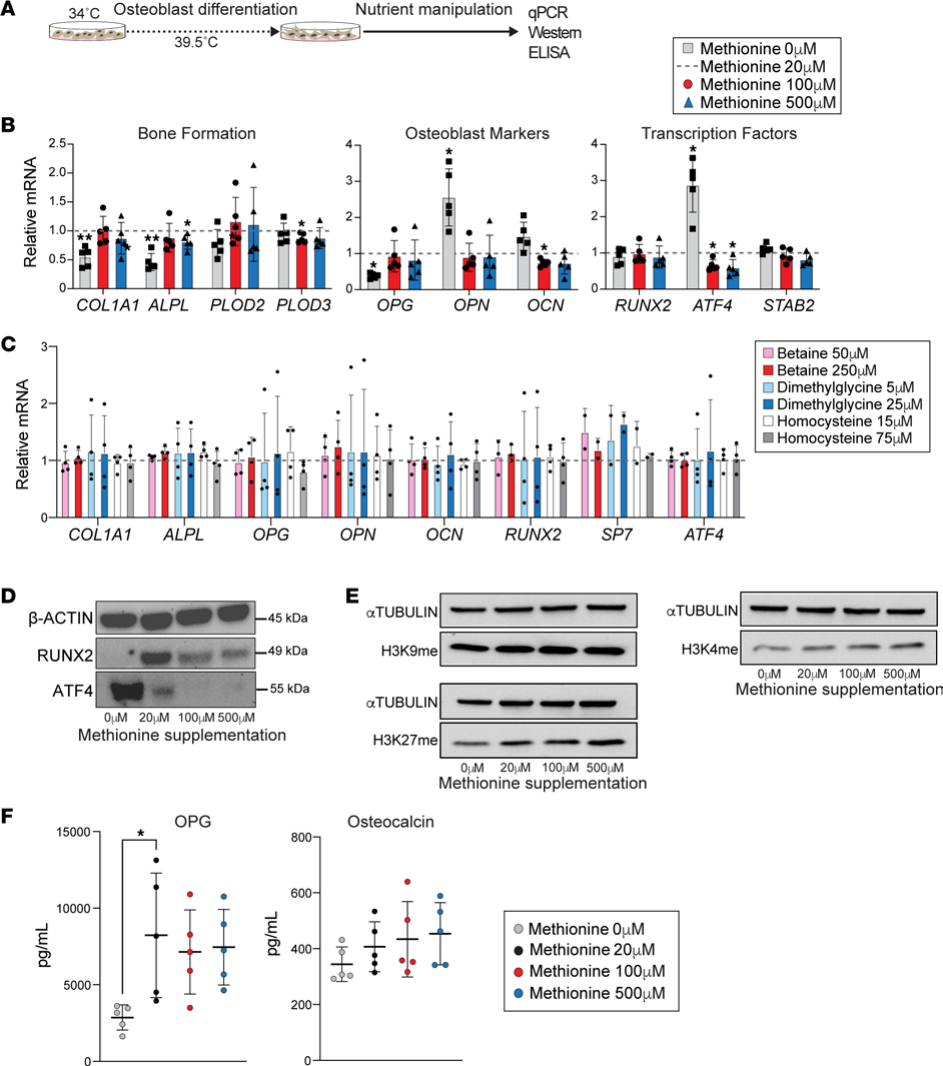
Cultured osteoblasts are sensitive to methionine concentration. (**A**) Experimental design. (**B**) Gene expression measured by qPCR of osteoblasts treated with no supplemental methionine or methionine excess (100 μM and 500 μM) for 48 hours. The mRNA expression of each gene was normalized to *GAPDH*. Data shown are relative to control (i.e., methionine 20 μM); **P < 0.01, 1-way ANOVA/Dunnett’s test for multiple comparisons (*n* = 5 biological replicates). (**C**) Gene expression measured by qPCR of osteoblasts treated with 2 high doses of betaine (50 μM and 250 μM), homocysteine (15 μM and 75 μM), or dimethylglycine (5 μM and 25 μM) for 48 hours. The mRNA expression of each gene was normalized to *GAPDH*. Data shown are relative to control (i.e., no treatment); *n* = 4 biological replicates. (**D**) Western blot analyses of osteoblasts exposed to similar ranges of methionine concentrations as in **B** and **C**, focused on 2 core transcription factors, RUNX2 and ATF4. (**E**) Western blot analyses of osteoblasts exposed to a similar range of methionine concentrations as in **B**, focused on chromatin methyl marks. (**F**) Bone formation markers measured by ELISA in the media of cultured osteoblasts exposed to variable methionine concentrations. *P < 0.05, 1-way ANOVA/Dunnett’s test.

We next performed a series of experiments in which we manipulated the levels of candidate one-carbon metabolites in cultured osteoblasts and examined expression of mediators of osteoblast cell state and function (Figure 3A). The standard hFOB1.19 cell culture medium was supplemented with several of the one-carbon metabolites, including methionine, glycine, and choline. Therefore, we first tested the effect of removing these metabolites from the media. We differentiated hFOB1.19 cells into mature osteoblasts (using regular media) and then switched to media without supplemented methionine, glycine, and/or choline, beyond that contained in the serum, for 48 hours (Supplemental Figure 2A). Methionine depletion, either alone or in combination with depletion of the other metabolites, resulted in differential expression of genes related to osteoblast specification or function, including *COL1A* (*P* = 0.08), *ALPL* (*P* = 0.453), *OPG* (*P* = 0.07), RUNX family transcription factor 2 (*RUNX2*) (*P* = 0.05), and activating transcription factor 4 (*ATF4*) (*P* = 0.07) (Supplemental Figure 2B). As such, we selected methionine for additional testing. We used a concentration of 20 μM as our physiological control, which is similar to circulating levels in healthy plasma (37), and compared gene expression with methionine depletion or 2 concentrations of methionine excess to model the directional change with fasting (100 μM, 500 μM). There was a significant reduction in transcription of genes related to bone formation (*P1NP* and *ALPL*, *P* < 0.05) and osteoblast markers (*OPG*, *P* < 0.01) with methionine depletion (Figure 3B). Expression of *ATF4*, a transcription factor that regulates osteoblast activity (38) and controls osteoblast differentiation toward the osteocyte lineage (39, 40), trended downward with excess methionine and was significantly upregulated (*P* < 0.01) with methionine depletion. Although not significant when sequential biological replicate experiments were merged, an interesting pattern was observed with *RUNX2*, another canonical transcription factor critical for osteoblast lineage specification, which trended downward with both methionine depletion and supplementation (Figure 3B).

The standard cell culture media is not supplemented with the one-carbon metabolites, homocysteine, betaine, or dimethylglycine, beyond that contained in the serum component. To examine potential effects of these metabolites on the same gene panel, we supplemented the media with ascending concentrations of each metabolite. In contrast with what we observed with methionine, mature osteoblast cultures did not demonstrate dose-dependent changes in osteoblast-related transcripts with homocysteine, betaine, or dimethylglycine (Figure 3C).

We additionally tested for methionine sensitivity of RUNX2 and ATF4 at the protein level, finding results that were directionally consistent with the gene expression data. Methionine depletion augmented ATF4 protein, and progressively increasing concentrations of methionine led to diminished ATF4 (Figure 3D). RUNX2 immunoblotting resulted in a pattern directionally consistent with transcriptional trends, as both methionine depletion and methionine excess reduced the RUNX2 signal. Given that methionine may supply methyl groups to methylate DNA, we examined histone methylation in mature osteoblasts cultured in different concentrations of methionine. By immunoblot, we found concentration-dependent effects on histone methylation, including methyl marks associated with transcriptional repression (Figure 3E). We also examined levels of bone formation markers in the media (Figure 3F), finding significant attenuation of OPG secretion with restriction of methionine, but no significant effect of methionine excess. Collectively, these data in cultured osteoblasts demonstrate variable effects of methionine concentration on regulatory markers of osteoblast cell state and function. A consistent theme was a greater response to methionine restriction relative to the modulatory effect of methionine supplementation. Changes in transcript and protein levels, particularly for in vitro models, may not replicate the in vivo context where multiple cell types are involved and/or the effects of variable metabolite exposures may be compounded over longer timescales; however, these experiments suggest that cultured osteoblasts exhibit sensitivity to methionine levels and that excess methionine may impair osteoblast cell state.

### Osteoblast differentiation is sensitive to methionine concentration.

**Figure 4:**
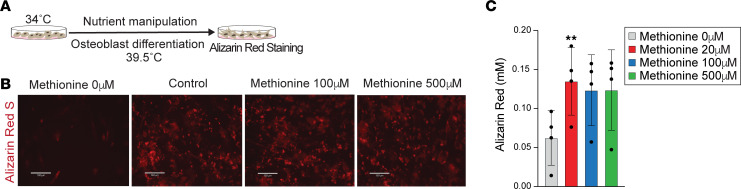
Osteoblast differentiation is sensitive to methionine concentration. (**A**) Experimental design. (**B**) Alizarin red S staining (scale bar = 130 μm). Cells were treated with no supplemental methionine or methionine excess (100 μM and 500 μM) when differentiation initiated, i.e., after transferring the cells to 39.5°C. (**C**) Alizarin red S staining quantification. Significance assessed relative to control; **P < 0.01, 1-way ANOVA/Dunnett’s multiple-comparison correction (*n* = 4 biological replicates).

Modulation of osteoblast differentiation may also affect bone homeostasis, and therefore we next manipulated methionine levels during the 16-day osteoblast differentiation protocol using a concentration range mirroring the mature osteoblast experiments (Supplemental Figure 3, A and B). When we removed supplemental methionine from the media, leaving only the methionine present in serum, and therefore a state of de facto methionine restriction, alizarin red staining was visibly reduced compared with control (20 μM of methionine) (Figure 4, A–C). By contrast, there was no visible reduction of mineralization matrix with excess methionine compared to control treatment (20 μM of methionine) (Figure 4B).

We also manipulated levels of other one-carbon metabolites, supplementing the media with betaine or homocysteine during osteoblast differentiation. No difference was detected in the mineralization matrix after 16 days of osteoblast differentiation with either betaine or homocysteine supplementation (Supplemental Figure 3, B and C). In demonstrating attenuation of osteoblast differentiation with methionine restriction, these data reinforce a role for methionine in osteoblast cell state; however, these experiments also reveal a complex interplay between methionine exposure and developmental timing. There may be an optimal window of methionine concentration, with potential negative consequences to bone biology with both methionine restriction (osteoblast differentiation) and methionine excess (maintenance of mature osteoblast cell state).

### Recapitulation of some aspects of the fasting response with methionine supplementation in mice.

**Figure 5:**
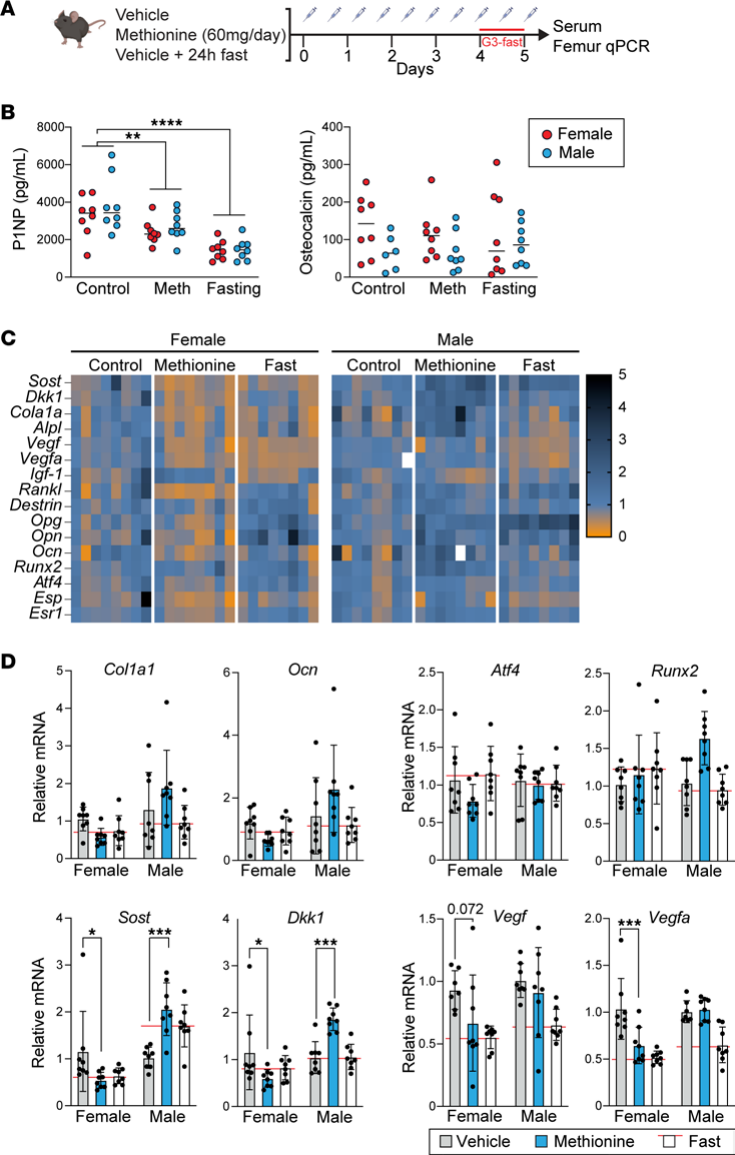
Recapitulation of some aspects of the fasting response with methionine supplementation in mice. (**A**) Experimental design. Male and female 14-week-old mice with a fasting group included as reference (*n* = 16 mice per group; *n* = 8 males and *n* = 8 females). G3, group 3. (**B**) Bone formation markers measured by ELISA. ****P < 0.001, **P < 0.01, 2-way ANOVA followed by Dunnett’s test for multiple comparisons with control. (**C**) Heatmaps of gene expression measured by qPCR of whole femur homogenates. The mRNA expression was normalized to Gapdh and vehicle control mice. (**D**) Key genes are shown as bar graphs with individual data points embedded in the graphs. Data shown are relative to control and separated by sex given dimorphic responses of some genes to both fasting and methionine. *P < 0.05, ***P < 0.001, 2-way ANOVA followed by Dunnett’s test for multiple comparisons.

We next considered the possibility that short-term methionine excess — similar in length to the human fasting methionine surge — would be sufficient to affect metrics of bone activity. To test this, we administered supplemental methionine (60 mg daily) or vehicle by intraperitoneal (I.P.) injection to female and male mice for 5 days. We also included a fasting group as reference to contextualize the scope and directionality of any observed changes (Figure 5A). We first examined circulating bone formation markers, finding a significant decrease in circulating P1NP levels (*P* < 0.05) with methionine administration relative to control mice (Figure 5B). The effect was directionally consistent with measurements in fasting mice, though the fasting effect was more pronounced (*P* < 0.05). While there was a trend toward reduction in circulating osteocalcin with methionine treatment or fasting, neither reached statistical significance (Figure 5B). Even though osteoblasts represent a minority of the heterogeneous bone tissue, we considered the possibility that bone gene expression might also be a sensitive indicator of any modifying effect of acute-subacute methionine excess on bone. Therefore, we performed qPCR on whole femur homogenates, focusing on a panel of genes involved in osteoblast cell state or anabolic bone metabolism. Even though the effects of fasting and methionine supplementation on bone turnover markers were similar between male and female mice (Figure 5B), we observed sex divergence of gene expression responses (Figure 5, C and D). For example, osteocyte genes involved in regulation of anabolic bone formation, sclerostin (*Sost*) and dickkopf WNT signaling pathway inhibitor 1 (*Dkk1*), were significantly modulated by methionine administration but in divergent directions. In female mice, expression of *Sost* and *Dkk1* significantly decreased (*P* < 0.05) with methionine treatment (Figure 5D), whereas both genes were augmented in male mice (*P* < 0.01) (Figure 5D). In the case of *Sost*, the methionine effect was similar in scope and direction to the fasting response. Vascular endothelial growth factors (VEGFs) promote bone formation in part by promoting angiogenic responses that support osteoblast activity (41, 42). Expression of *Vegfa* in the femurs of female mice decreased significantly with methionine treatment (*P* < 0.01), whereas a trend was seen in *Vegf* expression (*P* = 0.072) (Figure 5D). The osteoblast regulators that were methionine responsive in vitro, including *Runx2* and *Atf4*, did not demonstrate responsiveness to either fasting or methionine supplementation. The lack of a clear osteoblast signal, despite the effect of fasting and methionine administration on P1NP, may reflect the relatively small numbers of osteoblasts in whole bone relative to other critical cell types, while reflecting involvement of multiple cell types and potential modulating effects of sex.

Given the apparent effects of methionine excess on bone metrics in mice, we returned to the human data to test for associations between methionine and bone parameters. We used methionine area under the curve and change in methionine levels between baseline and achievement of peak levels and tested for correlations to trabecular parameters of radial bone microarchitecture, and to bone turnover markers, focusing on points when significant changes were noted. We found significant positive associations between percentage change in sclerostin between the baseline fasting day and day 7, percentage change in methionine from baseline to the end of the fast (*r* = 0.95, *P* = 0.004), methionine area under the curve (%) (*r* = 0.94, *P* = 0.005), and percentage change in methionine from baseline to achievement of peak level (*r* = 0.90, *P* = 0.01). Taken together, these murine and human data suggest that methionine levels may modulate bone metabolism; however, the in vivo data suggest a role for additional bone cell types, in particular the cellular source of sclerostin, the osteocyte.

## Discussion

Fasting is an increasingly popular approach to restriction of calories for weight loss and metabolic health. However, a well-documented detrimental effect associated with weight reduction is loss of bone mass, and consequently, increased risk of fracture. The time course and mechanisms underlying bone loss in the setting of fasting have not previously been well elucidated. In this study, we demonstrated that a 10-day, 0-calorie fast in healthy humans is sufficient to remodel bone microarchitecture and that elevated circulating methionine with fasting may contribute to bone loss.

Previous studies have demonstrated negative effects of fasting or calorie restriction on bone metabolism. In the most rigorous study of caloric restriction in humans, the CALERIE study, randomization of participants to 19.5% caloric restriction for 2 years resulted in significant loss of BMD at the lumbar spine, total hip, and femoral neck (14). Short-term fasting in humans (4 days) also showed a reduction in bone formation markers (11); however, the temporal dynamics of bone remodeling in response to fasting has not been extensively investigated. Indeed, studies describing bone loss following fasting or calorie restriction mostly use DXA (12–14). Although DXA is the most common clinical technique to assess BMD, it is relatively insensitive to short-term changes. HR-pQCT is a more sensitive technique that provides data on volumetric BMD and allows for the assessment of cortical and trabecular bone compartments. Indeed, a previous study demonstrated changes in trabecular bone with HR-pQCT over a 35-day protocol in which an overfeeding period preceded a fasting period (43). However, no prior studies have examined the effects of fasting alone on bone microarchitecture. Here, we detected no change in areal BMD with DXA at clinically relevant anatomical sites after a 10-day fast, whereas HR-pQCT demonstrated changes in BV/TV, Tb.N, Tb.Th, and Tb.Sp. Beyond demonstrating the power of HR-pQCT for short-term mechanistic and outcome studies, when the HR-pQCT data are considered together with the temporal dynamics of circulating bone anabolic markers — the decline in the marker of bone formation, P1NP, after 3 days of fasting — they indicate a window of 1–2 days from the onset of fasting when potential benefits of fasting may be incurred without bone loss. Previous studies have shown that fasting stimulates bone resorption. For example, the CALERIE study showed that long-term caloric restriction significantly increased levels of bone resorption markers, a finding visible after 6 months of caloric restriction (14). In our human study, we did not find a significant change in serum levels of CTX after fasting. However, considering the shorter 10-day time frame of our study, we cannot conclude that fasting does not impact bone resorption.

Although our data indicated deleterious effects of a prolonged fast on bone metabolism, trabecular thickness unexpectedly increased. This effect could be related to the coincident decrease in trabecular number. Indeed, studies in aging cohorts have shown similar results, as the trabecular compartment is characterized by age-related loss of thickness of horizontal trabeculae with no change in thickness of vertical trabeculae (44) — a phenomenon resulting in loss of trabecular number but increased trabecular thickness (44–48). We speculate therefore that trabecular number decreases with prolonged fasting and that trabecular thickness increases because the thinnest trabeculae (horizontal) are lost whereas the thicker trabeculae (vertical) remain intact. Furthermore, we observed a increase in the density of the cortical bone shell after 10 days of fasting, which is a surprising finding as caloric restriction is known to negatively affect cortical parameters, as shown in studies of anorexia nervosa (49). Our study does not provide an underlying mechanism for the cortical bone effect; however, a prior study investigating longer term (6-month) weight loss in overweight and obese men found a trend toward increased cortical thickness with weight loss (50). Despite the longer term design of the study by Pop et al. (50), both of our studies are relatively short-term studies with respect to weight loss, and it is possible that longer term weight loss is necessary to observe negative changes in the cortical compartment.

In this study, we identified dynamic modulation of one-carbon metabolic pathways with fasting, and we established methionine as a lead one-carbon metabolite candidate with regulatory properties in cultured osteoblasts. As an essential amino acid, methionine serves as an anabolic substrate for protein synthesis. Consistent with this principle, the removal of supplemental methionine from the media — leaving only methionine contained in the serum — inhibited osteoblast differentiation from cultured osteoblast precursors, consistent with the critical role of methionine metabolism for the maintenance and differentiation of human pluripotent stem cells (51). By contrast, methionine supplementation in fully differentiated osteoblasts suppressed osteoblast markers, including transcription factors important for osteoblast cell state, such as ATF4 and RUNX2. Even in the mature osteoblast, however, not all regulatory responses exhibited simple dose responsiveness. Whereas the stress-responsive ATF4 demonstrated dose-dependent repression across the full spectrum of methionine concentrations, RUNX2 was attenuated at both extremes of methionine exposure. The complex interplay between differentiation state and methionine levels likely indicates multiple underlying methionine-sensitive regulatory mechanisms. Beyond its function as an anabolic substrate, for example, methionine serves as a methyl donor and as such is involved in an array of intracellular biochemical pathways, including methylation of DNA/chromatin, nonhistone proteins, and different RNA species (52). Indeed, we demonstrate a global increase in H3K9me and H3K27me chromatin modifications in osteoblasts cultured with excess methionine, consistent with previously established links between methionine concentration and chromatin methylation states in other contexts, such as cancer cells and the liver (53).

In vivo studies in rodents and humans also demonstrate a complex relationship between the degree of methionine exposure and health outcomes. Like caloric restriction, long-term methionine restriction promotes longevity in model organisms, and the concomitant reduction in methionine may mediate some of the putative benefits of calorie restriction protocols (54–56). However, just as extreme caloric restriction, as seen in patients with anorexia nervosa, is pathological to bone and other tissues, extreme methionine restriction also negatively impacts bone metabolism, as murine studies have shown that bone formation markers (including P1NP) and bone mineralization significantly decrease following methionine restriction (57–59).

Mirroring our in vitro results, excess methionine exposure in mice and humans may also negatively impact metrics of bone metabolism. In an experiment designed to reflect the timescale of an acute fast in humans, we detected attenuation of P1NP and changes in transcripts related to osteoblast-osteocyte cell states in murine whole bone homogenates after 5 days of systemic methionine supplementation. While we also observed modulation of transcripts related to osteoblast-osteocyte cell states in murine whole bone homogenates, some responses to fasting and methionine diverged as a function of sex, consistent with well-recognized sex differences in skeletal mass and responses to caloric restriction (60) and methionine restriction (61, 62). Our data must be interpreted cautiously because of the challenge of deciphering the cellular source and the physiological significance of transcriptional signals from bulk analyses of heterogeneous tissues such as bone. However, a study that administered supplemental methionine to healthy human volunteers detected significant bone loss at the spine and hip (63). Therefore, our findings are consistent with an emerging theme that bone health is optimized within a methionine concentration window, an observation consistent with studies of other tissues, such as muscle cells and neurons (64).

Methionine is one of several amino acids that surge in circulation with a 0-calorie fast despite the absence of an exogenous amino acid source. One potential contributing factor is a general flux of intracellular amino acids into circulation, with skeletal muscle being an important tissue source (65). However, the surge in betaine — a substrate for conversion of homocysteine to methionine — coupled with upregulation of the enzymatic driver of this reaction in the liver (*Bhmt*) suggests that methionine recycling is also operative, which could explain the observed depletion of homocysteine coinciding with increases in betaine and methionine. Although our experiments with cultured human osteoblasts did not detect direct effects of homocysteine or other one-carbon metabolites beyond methionine, we cannot exclude regulatory functionalities for these factors in vivo or in relevant bone cell types that we did not study. It is notable, however, that it is generally elevation in homocysteine, rather than depletion as we observed with prolonged fasting, that predicts relevant bone pathophenotypes. Moreover, in monogenic hyperhomocysteinuria due to deficient cystathionine synthetase and characterized by low bone density, it is not just homocysteine but also methionine and other pathway members that are elevated (66). Indeed, the interconnectivity of the pathways that dictate systemic and local tissue concentrations of one-carbon metabolites underscores the challenge of determining whether specific metabolite predictors of disease phenotypes are causal pathological determinants. Without robust methods for cell type–specific control of methionine exposure, in vivo, in the way gene expression can be manipulated with tissue-specific genetic models, it will be challenging to definitively define the relationship between methionine levels and osteoblast function in vivo. Recently developed *Drosophila* models that enable genetic control over methionine levels in a tissue-specific manner may serve as a template for more definitive mechanistic rodent studies in the future (64).

In conclusion, our study demonstrates the power of HR-pQCT to detect acute remodeling of bone microarchitecture over 10 days of a 0-calorie fast and provides conceptual support for the fasting surge in methionine as a regulatory contributor. The interconnected nature of the metabolic pathways that determine methionine levels in circulation, coupled with the array of potential metabolic and molecular mechanisms that determine cellular responses to methionine, suggests that the regulation of osteoblast cell state by methionine as demonstrated here may be one of many ways that one-carbon metabolism impacts bone homeostasis. Emerging approaches to manipulate methionine metabolism in a cell type–specific manner in mouse models, coupled with multiomics characterization of regulatory programs in the heterogeneous bone microenvironment (e.g., single-cell transcriptomics and epigenomics), may elucidate the complex mechanisms that determine links between dietary content, circulating one-carbon metabolite concentrations, and bone responses, all of which will be critical to inform rational dietary prescriptions for optimization of bone health and aging more generally.

## Methods

### Sex as a biological variable

Our study examined male and female human participants. Our study also examined male and female mice, for which a subset of results suggested sex-dimorphic effects.

### Human participants

#### In-hospital fasting protocol.

We studied healthy participants who underwent a 10-day, 0-calorie fasting protocol as previously published (67). In this study, we include the data of 10 participants: 7 women and 3 men. Participants with a history of eating disorders or chronic conditions were excluded, and all women reported regular menstrual cycles. Participants first presented for a prebaseline visit, during which bone microarchitecture was measured using HR-pQCT and DXA was used to measure areal BMD. Ten days after the prebaseline visit, bone microarchitecture and areal BMD were remeasured. Participants were subsequently admitted to the Center for Clinical Investigation at the Brigham and Women’s Hospital for the 10-day fasting protocol, in which consumption was limited to daily multivitamin, 20 mEq potassium chloride, and 200 mg allopurinol, and water ad libitum. HR-pQCT and DXA were reassessed at the conclusion of the fast. Blood samples were collected at baseline (day 0), day 1, day 2, day 3, day 5, day 7, day 9, and day 10. For the baseline visit, blood was drawn after an overnight fast. One participant completed the prebaseline and baseline assessments but did not complete the fasting protocol, and a second participant completed the final assessments after 7 days of fasting.

#### DXA.

DXA scans were performed using a Discovery A densitometer (Hologic Inc.) to assess areal BMD. Participants were scanned at the posterior-anterior lumbar spine (L1–L4), lateral spine (L2–L4), left total hip, left femoral neck, and one-third distal radius. Coefficients of variation of DXA have been reported to be less than 2.2% for bone parameters (68).

#### HR-pQCT.

Trabecular and cortical bone density and microarchitecture at the distal radius and tibia were assessed with HR-pQCT (XtremeCT, Scanco Medical AG) as previously described (69, 70) in the nondominant arm or leg of the study participant. Short-term reproducibility, as assessed by repeat scans after repositioning 25 healthy study participants ages 20–30, ranged from 0.2% to 1.7% for density values and from 0.7% to 8.6% for microarchitecture variables, which is consistent with prior reports (71). One participant who completed the fasting protocol was a priori excluded from the HR-pQCT analysis because of incomplete HR-pQCT data at the 3 time points.

### Murine studies

WT C57BL/6J 14-week-old female and male mice (Jackson Laboratory) were housed under a 12-hour light/12-hour dark cycle at 22 ± 2°C with free access to water and food. Mice were randomized into 1 of the following groups: 1) I.P. vehicle injections for 5 days (negative control group; *n* = 8 female and *n* = 8 male), 2) I.P. vehicle injections for 5 days with a terminal 24-hour fast before euthanasia (fasting reference control; *n* = 8 female and *n* = 8 male), or 3) 60 mg/d excess methionine administered by twice-daily I.P. injections for 5 days; *n* = 8 female and *n* = 8 male.

### Plasma metabolites

Metabolomics for this study were previously published (21). The specific subset of metabolites reported in this manuscript were not previously shown, with the exception of methionine (contained in Figure 2), which we present again here as a critical component of one-carbon metabolism. The data set was also leveraged for new metabolic pathway analyses, using the Small Molecule Pathway Database (23, 24).

### Biochemical assays

Human serum P1NP was assessed by SimpleStep ELISA (Abcam ab210966) with a sensitivity of 5.3 pg/mL and an intra-assay coefficient of variation (CV) of 1.8% and interassay CV of 3%. Human CTX (plasma) was measured by ELISA (Novus Biologicals NBP2-82452) with a sensitivity of 75 pg/mL and intra-assay CV < 6.71% and interassay CV < 5.70%. Human serum PTH was measured by ELISA (Abcam ab230931) with a sensitivity of 0.761 pg/mL and an intra- and interassay CV of 1.8% and 3.8%, respectively. RANKL levels in human serum were assessed by ELISA (Abcam ab213841) with a sensitivity of <10 pg/mL and intra-assay CV of <6.2% and interassay CV of <7.4%. Human OPG was measured from serum by ELISA (Abcam ab100617) with a sensitivity of 1 pg/mL and intra- and interassay of <10% and <12%, respectively. Human serum OCN was assessed by ELISA (Abcam ab270202) with a sensitivity of 13.99 pg/mL and intraassay CV of 6.1% and interassay CV of 7.0%. Sclerostin levels in human serum were measured by ELISA (R&D Systems DSST00) with a sensitivity of 1.74 pg/mL and intra-assay CV of <2.1% and interassay CV of <10.8%. Mouse serum P1NP was measured by ELISA (Abcam ab210579) with a sensitivity of 6.7 pg/mL and intra- and interassay CV of 2.4% and 5.6%, respectively. Mouse serum OCN was assessed by ELISA (Novus Biologicals NBP2-68151) with a sensitivity of 0.23 ng/mL and intraassay CV of <7.69% and interassay CV of <7.14%. OCN levels were measured from cell culture media by ELISA (Abcam ab270202) with a sensitivity of 13.99 pg/mL and intra- and interassay CV of 6.1% and 7%, respectively. OPG levels in cell culture media were assessed by ELISA (Abcam ab189580) with a sensitivity of 11 pg/mL and intraassay CV of 6% and interassay CV of 9%.

### Osteoblast cell culture

The human fetal osteoblast cell line hFOB1.19 (ATCC, CRL-11372) was cultured in Ham’s F12 Medium DMEM, with 2.5 mM l-glutamine (without phenol red) media (Gibco), supplemented with 10% fetal bovine serum (Gibco) and 0.3 mg/mL of gentamicin at 34°C (5% CO_2_). For experiments in which specific one-carbon metabolites were restricted below the standard concentrations in the media, custom Ham’s F12 Medium DMEM without choline, glycine, and methionine was purchased from Boca Scientific. Although the hFOB1.19 line is a well-established preosteoblast model (35, 36), before conducting our in vitro experiments, we first revalidated its differentiation potential (Supplemental Figure 1), which guided selection of experimental time points. hFOB1.19 cells were plated at 1.5 × 10^5^ cells per well in 6-well plates, 4 × 10^4^ cells per well in 12-well plates, 1.0 × 10^6^ cells per 10 cm^2^ dish, or 3.0 × 10^6^ cells per 15 cm^2^ dishes depending on the experimental endpoint. Cells were grown to confluence at 34°C (5% CO_2_), then transferred to 39.5°C (5% CO_2_) to initiate differentiation. Media were changed every 3 days. Day 16 cells were used for mature osteoblast experiments.

### Alizarin red staining

Cultured cells were stained with alizarin red (ScienCell Research Laboratories 8678) according to the manufacturer’s protocol. Briefly, cells were washed with phosphate-buffered saline and fixed in 4% formaldehyde at room temperature for 15 minutes. Fixative was then removed, and cells were washed several times with deionized water (diH_2_O). Cells were then treated with 40 mM alizarin red solution for 30 minutes at room temperature with gentle shaking. Dye was washed several times with diH_2_O. Calcium deposits (red color) were visualized on a microscope (Echo, Revolve) and pictures were further taken. To quantify calcium deposits, 10% acetic acid was added the cells for 30 minutes (at room temperature). A cell scraper was used to collect the cells, and samples were heated at 85°C for 5 minutes, followed by 5 minutes of incubation on ice. Cells were then centrifuged for 15 minutes (20,000*g*). To neutralize the acid, 10% ammonium hydroxide was added to the samples. Staining concentration was then determined by absorbance (optical density at 405 nm) on a multiplate reader (Molecular Devices SpectraMax i3x).

### qPCR

Total RNA was extracted from cultured cells (hFOB1.19 cell line) using RNAzol RT (Molecular Research Center, Inc. RN190) and from murine tissues using TRIzol Reagent (Invitrogen 15596026). Briefly, cells or tissue specimens were placed in 1 mL of extraction reagent RNAzol RT, and RNA was extracted according to manufacturer’s protocol. cDNA was synthesized using the High-Capacity cDNA Reverse Transcription Kit (Applied Biosystems), and qPCR was performed using the PowerUp SYBR Green Master Mix (Applied Biosystems). Relative expression levels for each gene were normalized to the levels of GAPDH, and the ΔΔCt method was used to calculate fold-change. Primers used in the qPCR reactions are shown in Supplemental Table 2.

### Western blot

Cultured cells (hFOF1.19) were washed and lysed in RIPA buffer (Thermo Fisher Scientific), followed by sonication. Protein lysate concentrations were measured with DC protein assay (Bio-Rad), and equal protein amounts were loaded on a 10% Mini-Protean TGX Precast gel (Bio-Rad) followed by protein transfer to an Immun-Blot PVDF membrane on a semidry blotter (Bio-Rad). Detection of putative housekeeping proteins (tubulin and actin) was performed on separate membranes, rather than multiplexed analyses of a single membrane, because distinct bands for the target and housekeeping proteins were not resolved under these conditions. As such, quantification by densitometry was also not performed. Membranes were incubated in blocking buffer (1 hour at room temperature), followed by an overnight incubation (4°C) with the following antibodies: anti-Runx2 (1:1,000) (Cell Signaling Technology, RUNX2 D1L7F Rabbit mAb 12556), anti-ATF4 (1:1,000) (Cell Signaling Technology, ATF-4 D4B8 Rabbit mAb 11815), and anti–β-Actin (1:1,000) (Cell Signaling Technology, 13E5 Rabbit mAb 4970). An anti-rabbit IgG secondary antibody (1:5,000) (Cell Signaling Technology, Anti-rabbit IgG HRP-linked antibody 7074S) was further used to detect the target antigen. Western blot images were taken using a chemiluminescence detection imaging machine (Bio-Rad). For immunoblotting of total histone methyl marks, antibodies were obtained from EMD Millipore (H3K27me3, 07-449), Abcam (H3K9me, ab8898), and Sigma-Aldrich (Tubulin, T5168). Cells were lysed in RIPA (Cell Signaling Technology) lysis buffer. Whole-cell lysates were resolved by electrophoresis; proteins were transferred onto PVDF membranes (Immobilon P; MilliporeSigma), blocked in Tris-buffered saline/Tween 20 buffer (Cell Signaling Technology) containing 2.5% dry milk, and probed with the indicated antibodies in this buffer.

### Statistics

Statistical analyses on human data were performed using the JMP software, version 15 (SAS Institute). The GraphPad Prism software, version 9.5, was used to conduct statistical analysis on data from cell experiments. We report means and standard deviations unless the data were not normally distributed, in which case we report medians (interquartile range). Box plots show the interquartile range, median (line), and minimum and maximum (whiskers). To assess changes in bone microarchitecture from prebaseline to baseline and baseline to final fast day, paired *t* tests (2-tailed) were used if the data were distributed normally and paired Wilcoxon signed rank tests (2-tailed) if data were not normally distributed. Paired sample *t* tests (2-tailed) or paired Wilcoxon signed rank tests (2-tailed) were used to assess changes in circulating levels of P1NP, CTX, RANKL, PTH, OPG, OCN, and sclerostin. To analyze changes in plasma metabolites during fasting, we performed differential expression analysis comparing each fasting time point with baseline (day 0) and reporting *P* values adjusted for the multiple comparisons to baseline using the false discovery rate method as previously described (21). For in vitro experiments with more than 2 experimental groups, 1-way ANOVA was used with Dunnett’s test to account for multiple comparisons. For the murine studies, 2-way ANOVA was used with Dunnett’s test to account for multiple comparisons. Significance was set at *P* < 0.05.

### Study approval

The human study was performed at the Massachusetts General Hospital and Brigham and Women’s Hospital. The protocol was approved by the Mass General Brigham (formerly Partners) HealthCare Institutional Review Board. The protocol complied with the guidelines of the Health Insurance Portability and Accountability Act, and written informed consent was obtained from all participants. Animal experiments were approved by and in compliance with the University of Pittsburgh Institutional Animal Care and Use Committee.

### Data availability

Data from mice experiments and cell culture are all available in the Supporting Data Values file. Given the sample size of the human study, deidentified data from human studies will be made available upon reasonable request to the corresponding authors.

## Author contributions

TA designed experiments, participated in data acquisition, performed data analysis, and wrote the manuscript. WD, NGVK, NLD, TP, and NKD participated in data acquisition. BZ participated in data analysis. AP participated in data acquisition and data analysis. MLS and PKF designed experiments, participated in data acquisition, performed data analysis, wrote the manuscript, and supervised the study. TA, NGVK, NLD, WD, TP, NKD, BZ, AP, MLS, PKF participated in the critical review and approval of the manuscript.

## Supplementary Material

Supplemental data

Unedited blot and gel images

Supporting data values

## Figures and Tables

**Table 1:**
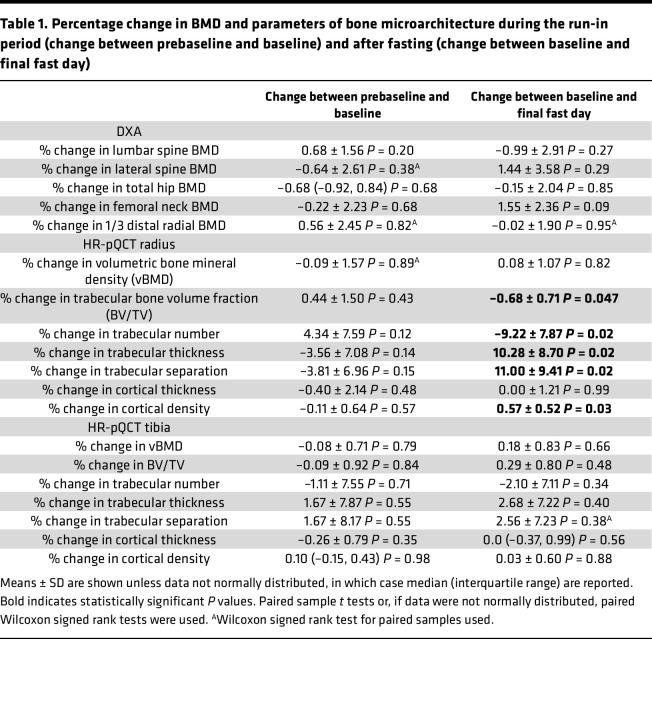
Percentage change in BMD and parameters of bone microarchitecture during the run-in period (change between prebaseline and baseline) and after fasting (change between baseline and final fast day)

## Version 1 05/23/2024

In-Press Preview

## Version 2 06/24/2024

Electronic publication
