# Supplementary material for: Methionine as a regulator of bone remodeling with fasting
Source: JCI Insight. 2024 May 21;9(12):e177997. doi: 10.1172/jci.insight.177997 (PMC11383369; doi:10.1172/jci.insight.177997)
Supplement: Supplemental data [file jciinsight-9-177997-s045.pdf]

Table S1: Characteristics of study participants at pre-baseline visit.

	**Females** (n=7)	**Males** (n=3)
**Age (years)**	28.2 [23.9, 39.8]	30.3 [21.8, 32.6]
**BMI (kg/m^2)**	26.5 [25.2, 28.3]	28.4 [27.8, 29.1]
**Bone mineral density**		
Lumbar spine (g/cm^2)	1.057 [0.930, 1.229]	1.079 [1.029, 1.188]
Lateral spine (g/cm^2)	0.884 [0.853, 0.893]	0.935 [0.833, 0.939]
Total hip (g/cm^2)	1.085 [0.983, 1.142]	1.126, [1.116, 1.227]
Femoral neck (g/cm^2)	0.901 [0.808, 0.979]	1.077 [1.030, 1.085]
Distal 1/3 radius (g/cm^2)	0.725 [0.710, 0.729]	0.826 [0.740, 0.911]

Median [interquartile range]

Table S2. Primer sequence.

**Human genes qPCR**	
**Genes**	**Primers**
COL1A1	Forward: GAGGGCCAAGACGAAGACATC
	Reverse: CAGATCACGTCATCGCACAAC
ALPL	Forward: ACTGGTACTCAGACAACGAGAT
	Reverse: ACGTCAATGTCCCTGATGTTATG
OPG	Forward: GTGTGCGAATGCAAGGAAGG
	Reverse: CCACTCCAAATCCAGGAGGG
OPN	Forward: GAAGTTTCGCAGACCTGACAT
	Reverse: GTATGCACCATTCAACTCCTCG
OCN	Forward: GGCGCTACCTGTATCAATGG
	Reverse: GTGGTCAGCCAACTCGTCA
ATF4	Forward: GTTCTCCAGCGACAAGGCTA
	Reverse: ATCCTGCTTGCTGTTGTTGG
RUNX2	Forward: TCAACGATCTGAGATTTGTGGG
	Reverse: GGGGAGGATTTGTGAAGACGG
STAB2	Forward: CATGCCACAGTCCGCAATG
	Reverse: GGCCCAGAACACAATAGTCTGA
OSTERIX	Forward: TTTGCTCCCCTTAATCCAGCC
	Reverse: CCTGGCAATTAGGGCAGTCG
CREB	Forward: AGTTTCAGCCGTCATTTCACC
	Reverse: AGCACTACCATCAAATTGTCGC
PLOD2	Forward: TTATTGAGCAACCAACCCCTTT
	Reverse: GGCTTCCGCTTGACTTAGATTT
PLOD3	Forward: CTGAAGAAGTTCGTCCAGAGTG
	Reverse: ACCGATGAATCCACCAGAATTG
BSP	Forward: CACTGGAGCCAATGCAGAAGA
	Reverse: TGGTGGGGTTGTAGGTTCAAA
**Mouse genes qPCR**	
**Genes**	**Primers**
Col1a1	Forward: GCTCCTCTTAGGGGCCACT

	Reverse: ATTGGGGACCCTTAGGCCAT
Alpl	Forward: GGCTGGAGATGGACAAATTCC
	Reverse: CCGAGTGGTAGTCACAATGCC
Sost	Forward: AGCCTTCAGGAATGATGCCAC
	Reverse: CTTTGGCGTCATAGGGATGGT
Dkk1	Forward: CTCATCAATTCCAACGCGATCA
	Reverse: GCCCTCATAGAGAACTCCCG
Dstn	Forward: GTTCAGGTTGCGGATGAAGTA
	Reverse: GCGACAATCTTTTTCAGGAAGC
Opg	Forward: CAGAGAAGCCACGCAAAAGTG
	Reverse: AGCTGTGTCTCCGTTTTATCCT
Opn	Forward: ATCTCACCATTCGGATGAGTCT
	Reverse: TGTAGGGACGATTGGAGTGAAA
Ocn	Forward: CTGACCTCACAGATCCCAAGC
	Reverse: TGGTCTGATAGCTCGTCACAAG
Runx2	Forward: AGAGTCAGATTACAGATCCCAGG
	Reverse: TGGCTCTTCTTACTGAGAGAGG
Atf4	Forward: TCCTGAACAGCGAAGTGTTG
	Reverse: ACCCATGAGGTTTCAAGTGC
Esp	Forward: GCCAGGTGGATTTGACTATGC
	Reverse: GACTCGTTGGTATGAGCTTGG
Igf-1	Forward: GTGGGGGCTCGTGTTTCTC
	Reverse: GATCACCGTGCAGTTTTCCA

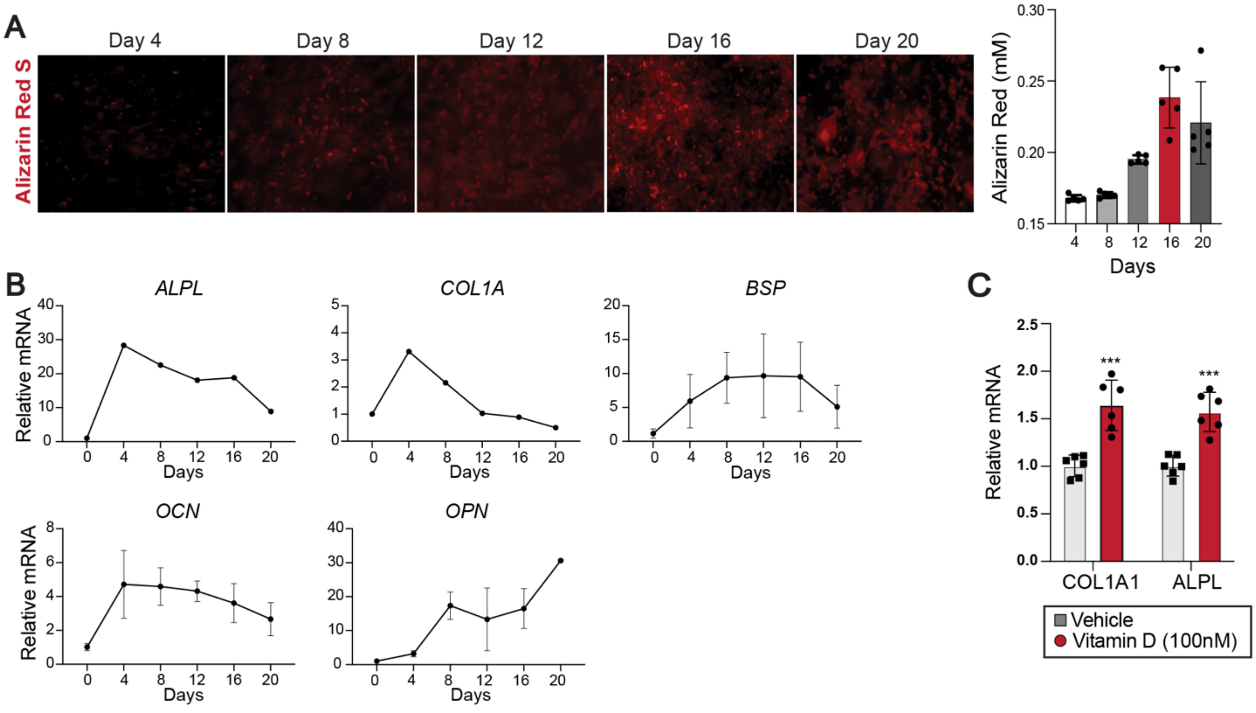

Figure S1. Human osteoblast model.

(A) Alizarin Red S Staining of differentiating hFOB1.19 cells. Cells were cultured at 34°C for 48 hours and then cultured at 39.5oC for 4, 8, 12, 16 and 20 days. Red staining shows calcium deposition; graph shows calcium concentration (mM) after 4, 8, 12, 16 and 20 days of differentiation.

(B) Gene expression measured by qPCR of immature osteoblasts (Day 0 - cells were cultured at 34oC for 48 hours), and in differentiating osteoblasts after transfer to 39.5oC. mRNA expression was normalized to GAPDH.

(C) Gene expression measured by qPCR after 16 days of differentiation at 39.5oC. Treatment with either vehicle or vitamin D (1α,25-Dihydroxyvitamin D3 - 100nM) was initiated for 48 hours. mRNA expression was normalized to GAPDH. Representative of two experiments; $***$ $p<0.001$, t-test (n=6 technical replicates), Shapiro-Wilk test $p>0.05$.

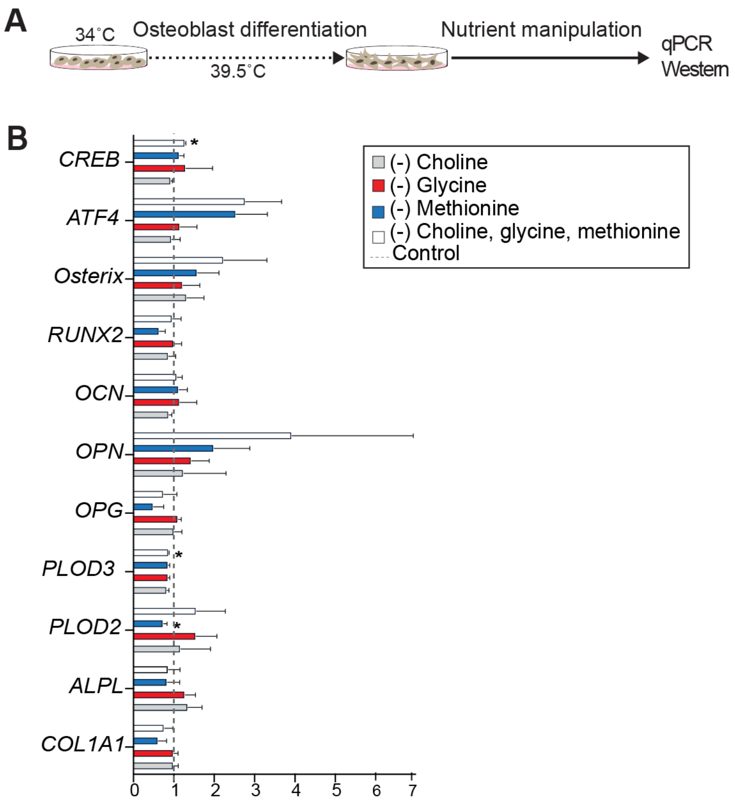

Figure S2. Osteoblast sensitivity to methionine depletion.
(A) Experimental design.
(B) Gene expression measured by qPCR. After 16 days of differentiation, choline, glycine and/or methionine were removed from the media for 48 hours leaving the 10%serum as the only source. mRNA expression was normalized to GAPDH; n=3 biological replicates, n=4 technical replicates.

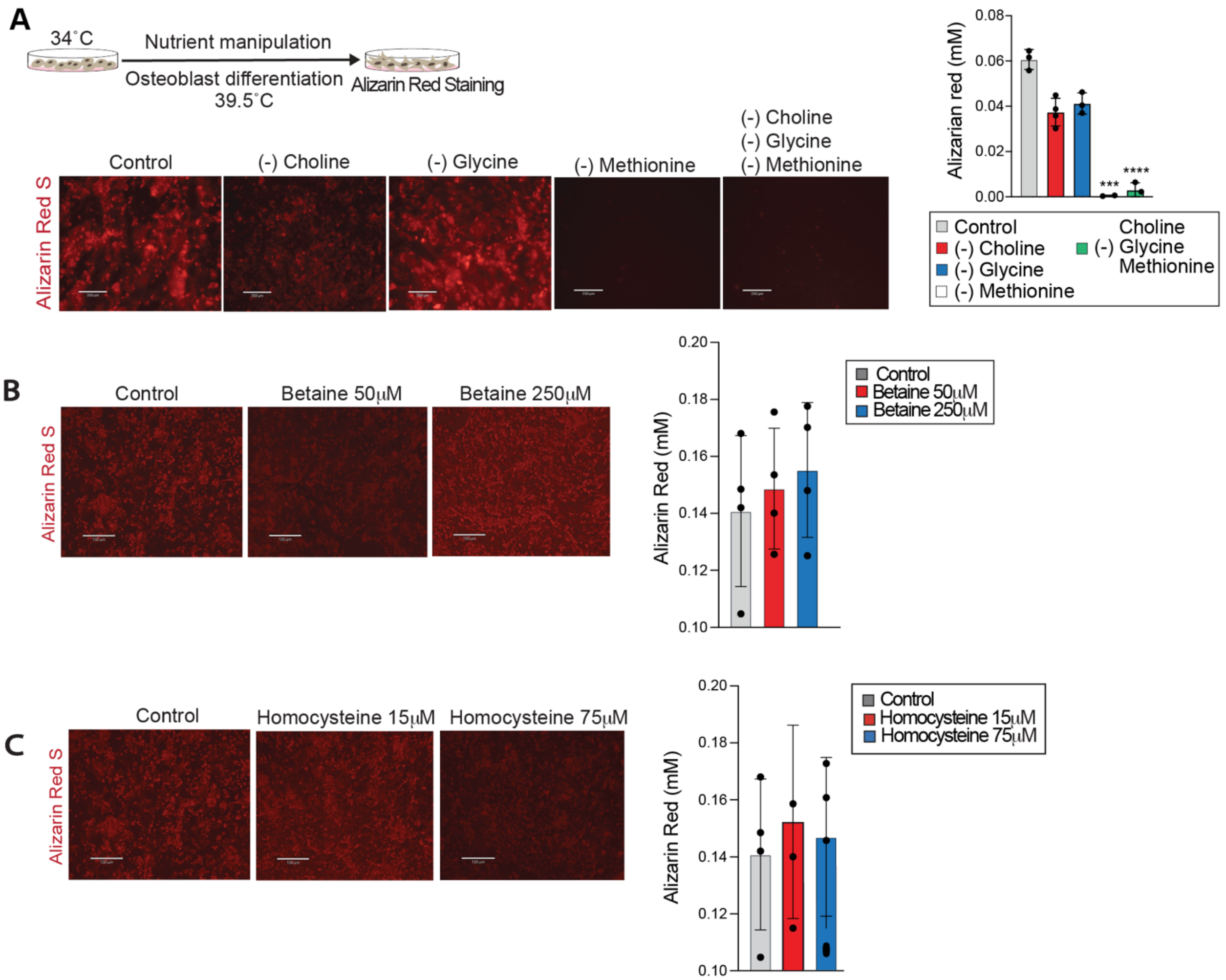

Figure S3. Osteoblast differentiation is sensitive to methionine depletion.

(A) Experimental design. Alizarin Red S Staining; scale bars=250 μm. Choline, glycine and/or methionine were removed from the media with initiation of differentiation, i.e., after transferring the cells to 39.5oC. Right graph: Alizarin Red S Staining quantification. Significance assessed relative to control; **p<0.01 One-way ANOVA, multiple comparison correction (3 techinical replicates).

(B) Alizarin Red S Staining; scale bars=130μm. Cells were cultured at 34oC for 48 hours and then cultured at 39.5oC for 16 days. Treatment with two doses of betaine (50μM and 250μM) was initiated with onset of differentiation, i.e., after transferring the cells to 39.5oC. Red staining shows calcium deposition; graph shows calcium concentration (mM).

(C) Alizarin Red S Staining; scale bars=130μm. Cells were cultured at 34oC for 48 hours and then cultured at 39.5oC for 16 days. Treatment with two high doses of excess homocysteine (15μM and 75μM) was initiated with onset of differentiation, i.e., after transferring the cells to 39.5oC. Red staining shows calcium deposition; graph shows calcium concentration (mM).
