# Supplementary material for: Methionine as a regulator of bone remodeling with fasting
Source: JCI Insight. 2024 May 21;9(12):e177997. doi: 10.1172/jci.insight.177997 (PMC11383369; doi:10.1172/jci.insight.177997)
Supplement: Unedited blot and gel images [file jciinsight-9-177997-s046.pdf]

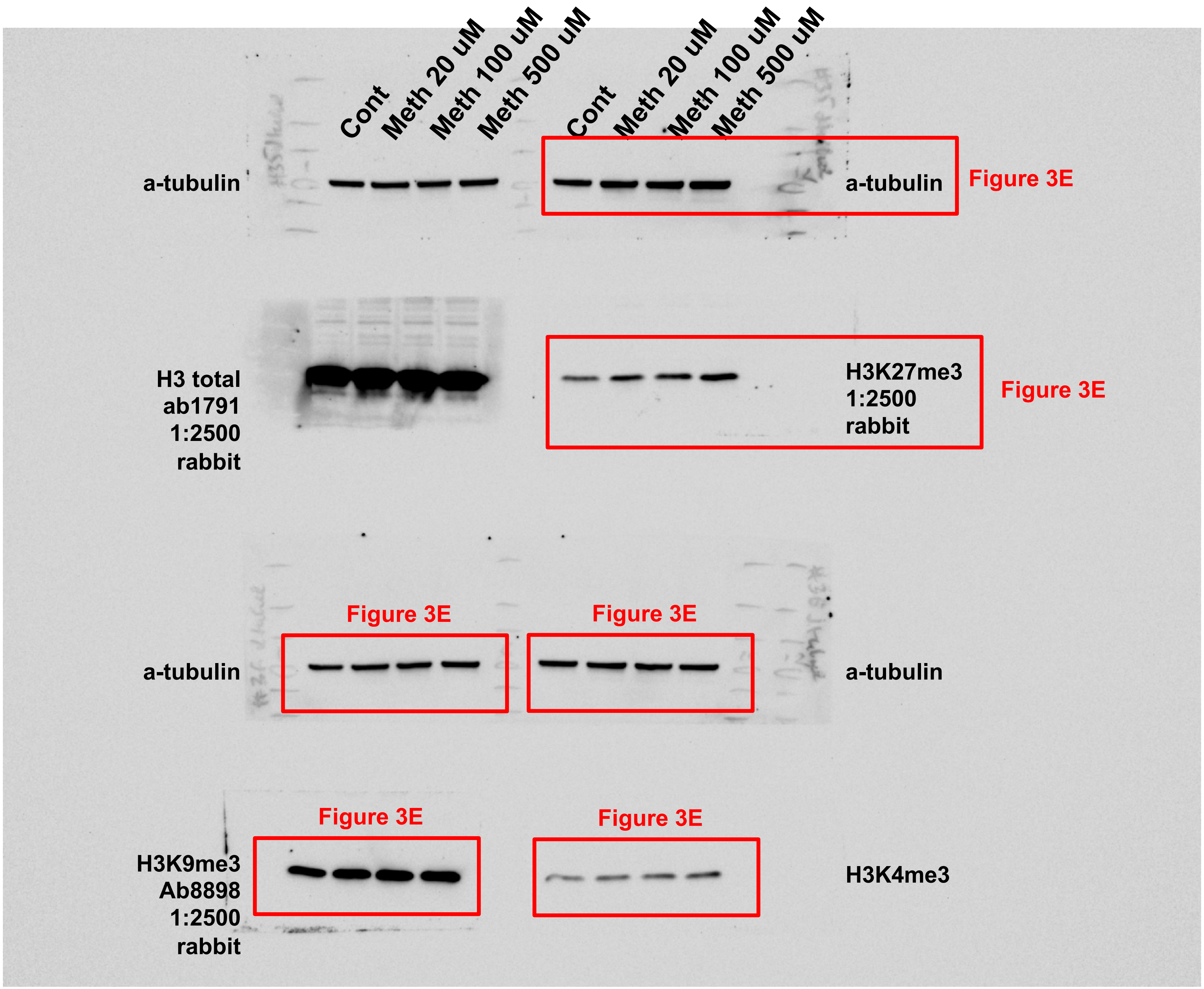
Cont
Meth 20 uM
Meth 100 uM
Meth 500 uM
Cont
Meth 20 uM
Meth 100 uM
Meth 500 uM
a-tubulin
a-tubulin
Figure 3E
H3 total
ab1791
1:2500
rabbit
H3K27me3
1:2500
rabbit
Figure 3E
Figure 3E
Figure 3E
a-tubulin
a-tubulin
Figure 3E
Figure 3E
H3K9me3
Ab8898
1:2500
rabbit
H3K4me3

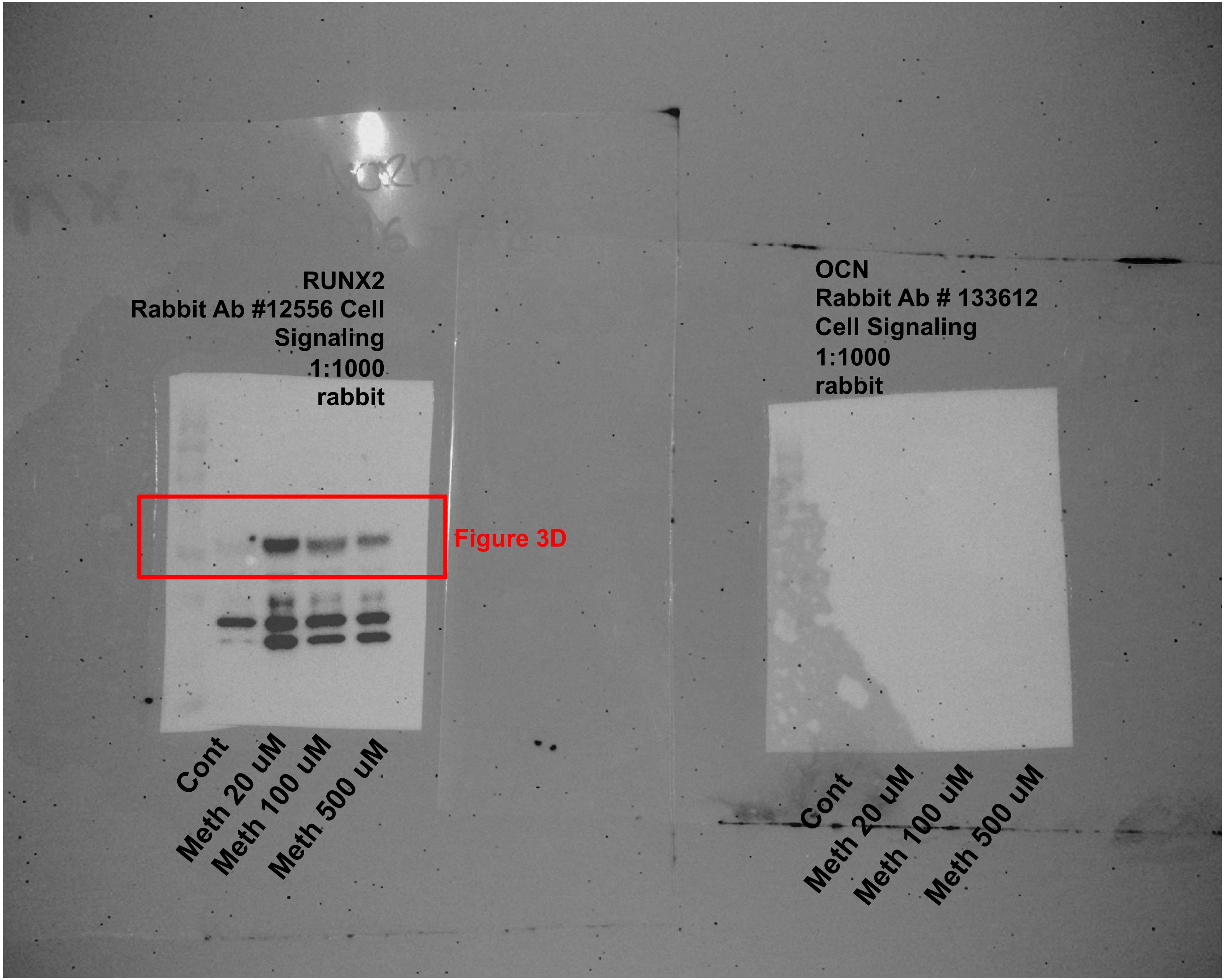
RUNX2
Rabbit Ab #12556 Cell
Signaling
1:1000
rabbit
Figure 3D
Cont
Meth 20 uM
Meth 100 uM
Meth 500 uM
OCN
Rabbit Ab # 133612
Cell Signaling
1:1000
rabbit
Cont
Meth 20 uM
Meth 100 uM
Meth 500 uM

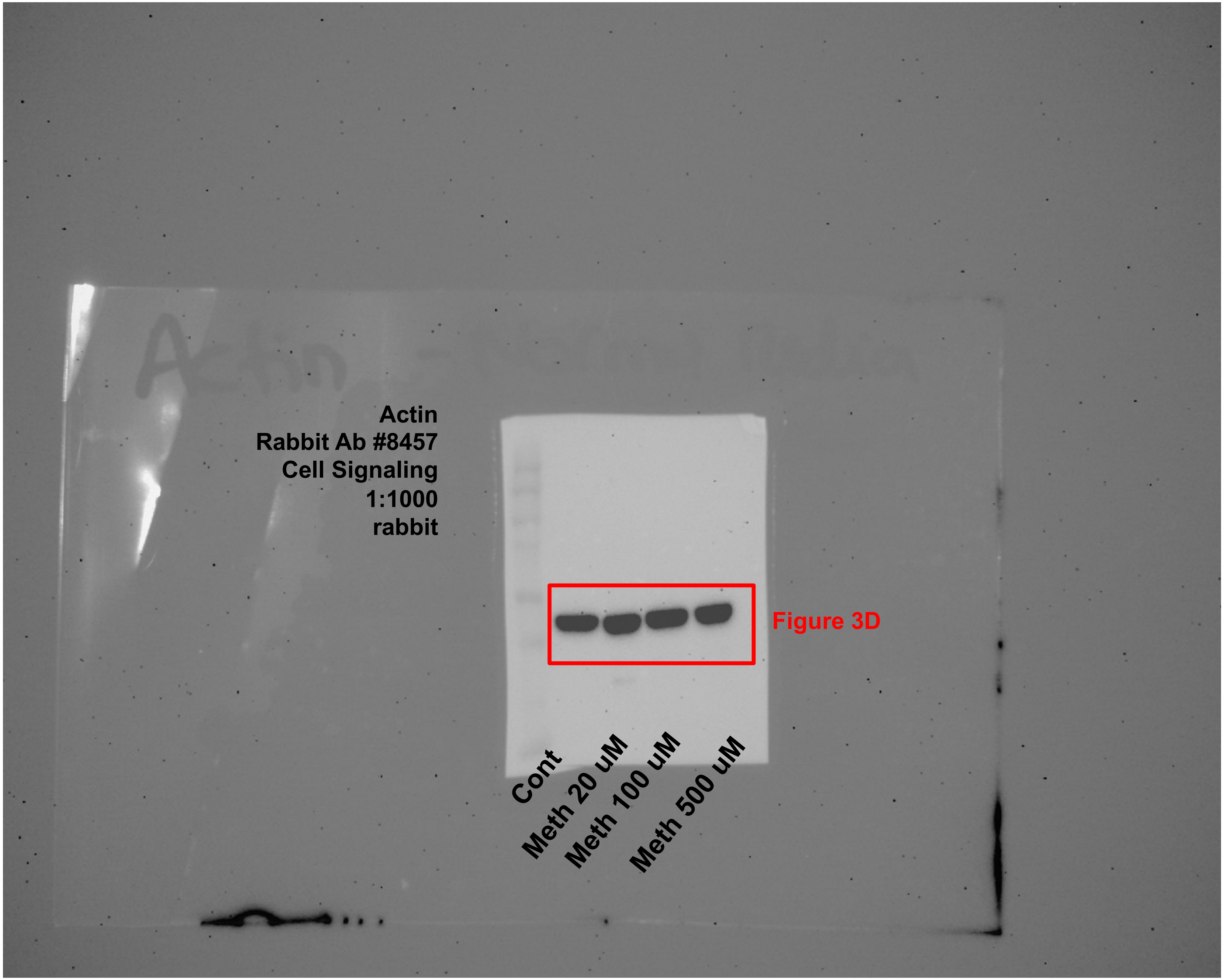
Actin
Rabbit Ab #8457
Cell Signaling
1:1000
rabbit
Figure 3D
Cont
Meth 20 uM
Meth 100 uM
Meth 500 uM

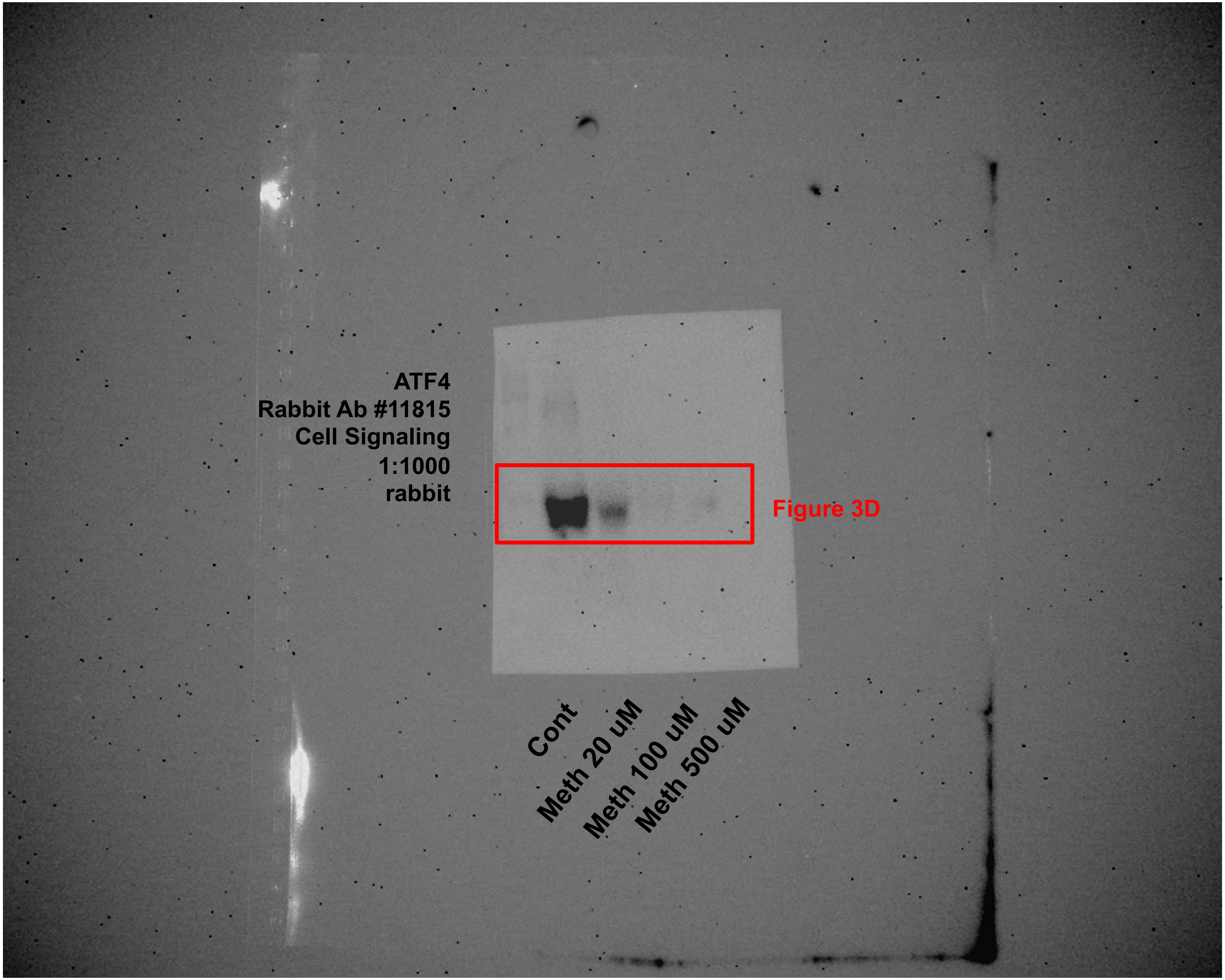
ATF4
Rabbit Ab #11815
Cell Signaling
1:1000
rabbit
Figure 3D
Cont
Meth 20 uM
Meth 100 uM
Meth 500 uM
